# Challenges and opportunities in neurophotonics discussed at the International Conference on Biophotonics 2017

**DOI:** 10.1117/1.NPh.5.4.040402

**Published:** 2018-10-30

**Authors:** Mark R. Hutchinson, Paul R. Stoddart, Anita Mahadevan-Jansen

**Affiliations:** University of Adelaide, ARC Centre of Excellence for Nanoscale Biophotonics (CNBP), Adelaide, Australia; Swinburne University of Technology, ARC Training Center in Biodevices, Melbourne, Australia; Vanderbilt University, Biophotonics Center and Department of Biomedical Engineering, Nashville, Tennessee, United States

**Keywords:** neurophotonics, pain, neurological disorders

## Abstract

Neurophotonics is an exploding field that spans the intersection of light and neurons for fundamental discovery and clinical translation. Optical technologies have significantly impacted brain research by probing into the mysteries of the brain, modulating brain activity, and improving patient care. Based on a discussion held at the International Conference on Biophotonics 2017, a group of leading researchers brainstormed to identify areas of unmet need in neuroscience and medicine, where biophotonics research could have the highest affect. We present two areas of future growth that spans basic research and clinical needs: management of chronic pain and interventional neuroimmunology. There are many directions within these areas that could be pursued for the ultimate goal of improved understanding of the brain and enhanced care of patients with neurological disorders.

## 1 Overview

Neurophotonics is a rapidly developing field that has seen tremendous interest around the world, from academia to industry and government to medicine. Funding for this area has grown nearly exponentially and publications routinely use this phrase, where none existed 20 years ago. However, the definition of neurophotonics has been interpreted in different ways by many groups. These include “the use of photonics to study the brain and its processes at the cellular and molecular level,”^1^ “harnessing the power of light to study the nervous system and understand the mysteries of the brain,”^2^ and “the use of microscopic and spectroscopic methods in neuroscience,”^3^ among others. Most of these definitions seek to characterize this field as being at the intersection of optics and neuroscience. While most refer to the use of photons to understand the brain and its processes at the cellular and molecular level, in reality, this field broadly encompasses the application of light in all aspects of the brain and nervous system, i.e., the interaction of photons and neurons. This area of research has employed photons to (1) interrogate the cellular processes of the peripheral and central nervous systems (CNS) to understand the brain, (2) manipulate neurons to modulate function, and (3) detect disease for clinical diagnosis and surgical guidance. This transdisciplinary field bridges the disciplines of optical physics, chemistry, biomedical engineering, neuroscience, and neurosurgery, and has resulted in the creative use of optical technologies for both fundamental discovery and clinical translation. The field employs a range of optical methodologies, from microscopies to spectroscopies, to achieve a multiscale understanding of the structure and function of normal and diseased brain as well as the nervous system.

The United States government created the BRAIN initiative, which seeks to “deepen understanding of the inner workings of the human mind and to improve how we treat, prevent, and cure disorders of the brain.”^4^ Much of the recent efforts have gone toward achieving the first of the two goals of the initiative—understanding the brain. To match that effort, the European Human Brain Project strives to accelerate research at the intersection of neuroscience, computing, and brain-related medicine.^5^ These large-scale funding programs have allowed the blooming of extensive research that pushes the boundaries of what we know about the brain. Biophotonics has played a key role in this growing body of work. With its ability to probe at the atomic, molecular, cellular, tissue, and organ level, the interaction of light with the nervous system has allowed new directions in brain research and the birth of neurophotonics. There has been a growth in the past 15 years of publications at the intersection of light and the neurosciences but the rate of activity appears to have plateaued in this field. And yet, the premier conference in the field of neuroscience, the annual meeting of the Society of Neuroscience has seen a tremendous rise in the number of papers that include optical techniques, indicating the acceptance of the power of optics and photonics in neuroscience research. However, there remains much in the area of neural research that is untapped and many challenges in the care of neurological disorders that need to be addressed.

During the 2017 meeting of the International Conference on Biophotonics (ICOB) in Perth, Australia, a team of scientists gathered to discuss the current state of neurophotonics research and the challenges and opportunities that are available to the field. The group sought to identify future areas of growth that seek biophotonics solutions.

## 2 Current State of Neurophotonics

With the investments in brain research starting well before the formulation of the US BRAIN initiative and the European Human Brain Project, the field has made great strides in the three main areas of neurophotonics: (a) improving our understanding of how the brain works, (b) modulating the nervous system for precision control, and (c) detecting disease toward improved diagnosis and therapy. Confocal microscopy, multiphoton microscopy, fluorescence lifetime imaging, and many such techniques in conjunction with molecular probes have allowed researchers to track neural activity at the molecular level with spectacular images providing a window into the relevant processes and interesting insight into the structure and function of the brain.^6^ New optical techniques, such as swept confocally aligned planar excitation (SCAPE) microscopy, have risen from the hunt to see smaller, faster, deeper, and in 3-D.^7^ New targeted contrast agents are being developed to visualize the dynamics of specific events and genetically modified approaches are changing the way we approach this area of research.^8^ Various optical approaches have also been developed to modulate the nervous system. Several reviews can be found presenting the state-of-the-art of optogenetics, a technique that relies on genetically engineered cells, which express a light-sensitive opsin that may be turned on or off with unprecedented precision.^9^^,^^10^ This approach has been used to study neural networks in cultured neurons as well as *in vivo* mouse brain.

Infrared neural modulation on the other hand relies on a label-free approach to excite or inhibit neural activity and has been applied toward the development of novel cochlear implants as well as nerve monitoring in patients *in vivo*.^11^^,^^12^ New hybrid approaches portend to an exciting future for the modulation and control of the nervous system that has the potential to realize integrated human–machine interfaces.^13^ Optical approaches have also paved the way for the diagnosis and surgical guidance of brain disease. While the majority of this research seek to apply various spectroscopic methods to improve patient outcome following brain tumor resection,^14^^,^^15^ research on early detection of Alzheimer’s disease indicates the potential of light-based methods for improved patient care.^16^

Clearly, this paper is not meant to provide a review of the state-of-the-art in the various areas of neurophotonics and does not do justice to all of the various research efforts that are ongoing in this field. However, it does emphasize some topical directions of neurophotonics research and opens the door to new opportunities for current and future researchers in this field.

## 3 Future Opportunities in Neurophotonics

The group of scientists gathered together at ICOB identified two main areas of unmet need—neuroimmunology and chronic pain, where biophotonics research could have a tremendous impact. The rest of this paper presents a summary of the discussion that ensued and seeks to communicate the excitement and outlook for these areas of growth.

### 3.1 Probing the Brain without Probing the Brain

At the turn of the millennium, a sea-change occurred in neuroscience research owing to the acknowledgement of key bidirectional signaling that occurs between neurons and a host of immunocompetent cells present in the CNS, including glia (microglia, astrocytes, and oligodendrocytes), endothelial cells, perivascular macrophages, and infiltrating peripheral immune cells.^17^ Once thought of as just “brain glue” (glia) or excluded from CNS cellular anatomy (peripheral immune cells), these cells are now known to be far more than passive bystanders or components of the extracellular matrix, and have been shown to modulate neurotransmission within the CNS.^18^ Such a relationship may be best described as the neuroimmune interface.^19^ This anatomical unit, in its simplest form, is replicated billions of times throughout the CNS and comprised of neuronal processes (pre- and postsynaptic), astrocytic projections, and microglial surveillance, possibly with selective migration of peripheral immune cells to unique cellular events.

Therefore, the CNS cannot be assumed to be an immune-privileged organ. Instead, the brain and spinal cord are immune-active, receiving and contributing signals from the peripheral immune system. This bidirectional communication between peripheral and central immune systems presents us with the unique opportunity to sample peripheral cellular immune functions to understand CNS health and disease. And as knowledge of this relationship develops, defined information about specific CNS functions will be obtained. It is proposed that by using advanced biophotonic methodologies to approach these peripheral compartments, an indirect-neurophotonics information pipeline and biological manipulation method will be possible. The modular units of this interface will be introduced below, and how they contribute to the ability to monitor the brain from outside the brain may be elucidated.

The importance of glial contributions to health and disease of the brain and spinal cord has developed out of an appreciation of immune signaling within the CNS. Early work regarding the behavioral adaptations following systemic cytokine exposure helped to consolidate the field in its appreciation of these soluble factors.^20^ Although in the advanced illness response behavior studies, the endogenous origin of most immune factors and their sites of action that triggered behavioral changes remained unclear for some time. It was not until the aforementioned characterization of glial reactivity states that the power of glial responses was appreciated.^21^ However, the nature of immune signaling within the normal healthy brain remains unexamined owing to the scarcity of the signal events, and the tightly controlled timeframe over which they are allowed to act.

The peripheral immune system is a hunting ground for biomarkers of CNS function.

Given the recognition that there is bidirectional communication between brain/spinal and peripheral immune cells, there has been an exploration of the hypothesis that peripheral immune cells mirror the activity of the spinal immune and brain immune mechanisms that maintain the health and disease states of the CNS.^22^ It has been established that brain and spinal neuroimmune mechanisms rely on support of peripheral immune cells and that the *ex vivo* activity of peripheral immune cells can stratify patients into disease and healthy populations.^23^ These exciting data have implications for the field of neurophotonics, as light-based methods provide the opportunity to study the brain and its processes at the cellular and molecular level using peripheral immune cells without entering the CNS.

Such developments in remote-neurophotonics will rely on advances in rapid peripheral immune cell phenotyping using advanced biophotonics approaches and associated big data deep learning analytical techniques. Issues relating to the sensitivity and specificity of these diagnostic tests will need to be explored in prospective clinical trials across multiple populations. Additionally, a growth in the appreciation of the neuroimmune and immune-brain axis will be needed. A short description of two key immunocompetent cell types of the CNS is included here to aid in the understanding of the evolving view of the brain as an immune-competent organ.

#### 3.1.1 Astrocytes

Within the neuroimmune interface, the majority of research has focused on the role of astrocytes and microglia and, to a lesser extent, on that of T cells and endothelial cells. Astrocytes are the most abundant cell type in the CNS. In addition to providing structural support, promoting formation of the blood–CNS barrier and regulating cerebral blood flow, astrocytes contribute to synaptic transmission, provide trophic support, and promote repair of neuronal systems. They also maintain homeostasis in the extracellular environment by regulating the concentration of neurotransmitters and ions in the synaptic cleft. Their morphology and functions are highly polarized and heterogeneous.^24^

#### 3.1.2 Microglia

Microglia are the tissue-specific phagocytes of the CNS. They exhibit constitutive and regional heterogeneity throughout the parenchyma, presumably to co-ordinate diverse responses to insult, or in dynamic response to the microenvironment of their local neuroimmune interface. Under a basal surveillance state, the cyto-architecture of microglia allows them to continuously sample the extracellular space for perturbations. Transition to a state of reactive gliosis involves changes in cell number, morphology, phenotype and motility, the expression of membrane-bound and intracellular signaling proteins (for example, mitogen-activated protein kinases), and the release of immunoregulatory products, such as cytokines and chemokines.^25^

We therefore propose that study of the remote biological components may provide a window into the behavior and response of the CNS and can be used to mediate human response. For example, study of the breath exhaled in a patient with traumatic brain injury may provide insight into the severity of the injury. Urine may contain biomarkers that could indicate the health of the endocrine system. And exosomes such as vesicles may carry signals indicative of disease in the brain that should be explored. This concept of seeking a connection between the primary unit by studying the periphery is not new. Researchers have proposed that biomarkers of remote cancers exist in the blood plasma and can be used to identify the early onset of disease.^26^ Study of various accessible biological components, such as odor, breath, urine, blood, sweat or saliva, in relation to specific neurological disorders, may provide a window to the neuroimmune coupling of the human body and a more readily accessible target of research.

### 3.2 Neurophotonics for Pain Management

Chronic pain is Australia’s fourth most prevalent health condition.^27^^,^^28^ It is estimated that 20% of American adults suffer from pain that disrupts their sleep^29^ and back pain is the leading cause of disability in Americans under 45 years of age.^30^ Thus, globally, chronic pain is a major clinical problem with high morbidity and enormous social and economic cost.^31^ Pain costs each country millions to billions of dollars due to increased health care costs, rehabilitation costs, loss of income and productivity, and insurance coverage. Despite its impact, there is no clear approach for detecting pain, and treatment modalities are desperately needed. The past 15 years has seen a rapid growth of interest in optical techniques for interfacing with the nervous system, including methods, such as optogenetics and infrared neural modulation.^32^

In the context of infrared neural modulation, the ability to thermally inhibit neural activity with high spatial precision has generated interest in using the technique to block pain signals.^33^ The dorsal root is an attractive target for this application, as it is already addressed by implanted dorsal root stimulators. However, the current electrical stimulators have a general numbing effect and are limited from 8- to 9-year lifetime before replacement. It is anticipated that infrared neural modulation could reduce the side effects of these devices through improved spatial precision.

Despite this promise, a series of challenges will need to be addressed. Thermal inhibition requires a transient temperature rise of ∼5°C.^33^ For safe operation over extended periods, it is necessary to identify an appropriate duty cycle for the inhibitory stimulus. It may be possible to reduce the temperature increment by using an array of phase-delayed light sources spaced along the nerve trunk to generate a cumulative damping of the nerve signals. However, further work is required to determine the most effective means for light delivery. For example, there are no LEDs currently available at 1870 nm, so the power budget of a laser-based implant might be prohibitive.

As a further alternative, infrared heating might be combined with localized cooling to prevent tissue damage. While an interesting suggestion, this raises further questions about power budgets, optimum locations, and physiological responses, all of which are difficult to answer at this stage. The specific targeting of the IR pain treatment stimulus to afferent versus efferent pathways to enable the best outcomes also needs to be further explored. It was recognized that unmyelinated C-fibers may be more sensitive to photothermal inhibition and may provide a preferred target. In general, beyond possible protection from inflammation, there appears to be a lack of knowledge about the role of glial cells/astrocytes in pain pathways, as noted in the previous section.

Clearly, a great deal of preclinical research will be required to establish this treatment modality as one worthy of translating to humans. As such, a valid and translationally relevant preclinical model of persistent pain needs to be validated. There is a clear need for biophotonics experts to work with physiologists to identify the most promising approaches. Alternative approaches which also rely on neurophotonics include optogenetics^9^ and nanoparticle mediated technologies.^34^ There are significant concerns about regulatory challenges and public acceptance for both optogenetics- and nanoparticle-enhanced infrared modulation. While it is hard to predict how these concerns will be addressed in future, it is anticipated that nanoparticle-based approaches may prove to be more acceptable to patients, provided that exhaustive toxicology studies are conducted and show negligible side effects.

The relationship between infrared neural modulation and low-level light therapy (LLLT) was also raised. It is known that low levels of light can stimulate the growth of stem cells.^35^^,^^36^ There are limited systematic studies of the effects of light on CNS cells and, specifically, no quality studies of the optical properties of neural tissue *in vivo*. Overall, there are very limited data about the sensitivity of living tissue to different wavelengths of light. We do not know what chromophores are present at trace levels. For example, it is known that visible light illumination of riboflavin leads to the production of reactive oxygen species,^37^ which might in turn have an effect on the immune response. How this may be used to engender a therapeutic effect remains to be explored. Less invasive techniques may also be worth considering. Drugs have long been the hallmark of pain therapies. However, light-based approaches such as LLLT are being explored as effective analgesics^38^ and the role of the neuroimmune pathways in pain remains to be studied. Understanding the basis of pain and developing new biomarkers/targets for therapy remains an unexplored goal. Furthermore, there is some evidence that transcutaneous electrical stimulation of the auricular branch of the vagus nerve can alleviate depression.^39^ Since light provides a more precise and selective approach to stimulation, it may be speculated that selective wavelengths of light may be used to yield a similar effect. Relatively unexplored is the area of quantifying and detecting pain.

## 4 Uncharted Territories in Neurophotonics

Here, we have presented two potential areas of neurophotonics research that may have a tremendous impact on health care. While the proposed areas of research may seem to be too far-fetched, it should be noted that the field of biophotonics has historically relied on the seemingly challenging questions and approaches for its impact. It should be noted that accessing the human brain in live individuals is difficult using optical means, and recent research in neuroimmunology presents a potential vehicle for probing the brain. On the other hand, chronic pain is a huge burden on our economy and patients with chronic pain are desperate for solutions. Both neuroimmune responses, and direct and indirect light-based methods may aid in identifying solutions to this unmet need. It should be noted that there are certainly many more challenges than were identified by the ICOB scientists. In fact, many of the most common brain disorders, such as psychological conditions, remain uncharted territories in biophotonics research. We anticipate that this report is only the beginning of addressing the challenges that we face in this area, which are also the opportunities for the future.
